# Threat prioritization and fear of pain dynamics are associated with engagement in postoperative activities after thoracoscopic lung surgery

**DOI:** 10.1038/s41598-026-46036-5

**Published:** 2026-04-02

**Authors:** Yang Luo, Jiao Peng, Lijuan Feng, Lihong Bao, Xiao Wu

**Affiliations:** 1 Department of Thoracic Surgery, Union Hospital, Tongji Medical College, Huazhong University of Science and Technology, Wuhan, China; 2 Department of Respiratory and Critical Care Medicine, Wuhan Fourth Hospital, Wuhan, Hubei Province China; 3 Department of Nursing, Union Hospital, Tongji Medical College, Huazhong University of Science and Technology, Wuhan, China

**Keywords:** postoperative fear of pain, threat prioritization, avoidance behaviors, lung surgery, postoperative recovery, Psychology, Patient education, Pain

## Abstract

Fear of pain (FOP) often leads to activity avoidance, hindering recovery after thoracic surgery. We proposed the concept of “Threat Prioritization”—the cognitive weighing of activity-induced pain against inactivity-induced complications. This study investigated whether prioritizing complication risks over pain is associated with greater engagement in postoperative activities and a subsequent reduction in FOP. In this prospective cohort study, 121 patients undergoing thoracoscopic lung surgery were enrolled. FOP (Fear of Pain Questionnaire-9) and cough strength (Semi-Quantitative Cough Strength Score, SCSS) were assessed on postoperative days (POD) 1 and 3. Therapeutic ambulation episodes (POD1–3) were recorded. On POD3, patients were categorized as “Complication-Focused” or “Pain-Focused” based on their self-reported primary concern during recovery. Multiple linear regression was used to identify predictors of FOP reduction. Complication-Focused patients (*n* = 45) showed significantly stronger cough on POD3 (Median SCSS: 4.00 vs. 2.50, *p* < 0.001) and higher distribution of ambulation episodes (Median: 4.00 vs. 4.00, *p* = 0.003) compared to Pain-Focused patients (*n* = 76). Postoperative air leak rates were comparable between groups (20.0% vs. 19.7%, *p* = 1.000). Regression analysis (Adjusted R^2^ = 0.433) showed that greater FOP reduction was associated with higher ambulation counts (B = -1.25, *p* < 0.001). Conversely, activity-linked breakthrough pain was associated with attenuated fear reduction (B = 5.31, *p* < 0.001). Changes in physiological pain intensity were not significantly associated with FOP reduction (*p* = 0.964). Age, sex, and surgical extent were included in the model a priori but were not significant predictors (all *p* > 0.05). A mindset prioritizing complication risks over pain is associated with increased engagement in essential recovery activities without increasing observed air leak rates. Postoperative FOP reduction appears to be driven by behavioral engagement rather than physiological pain relief alone. These findings suggest that perioperative interventions targeting threat prioritization may facilitate functional recovery.

## Introduction

Fear of pain (FOP) is highly prevalent among perioperative patients, affecting up to 88.8%^1^. FOP hinders postoperative functional recovery by amplifying perceived pain severity and delaying essential early activities^2,3^. According to the fear-avoidance model, this phenomenon stems from a catastrophic misinterpretation of pain as a signal of serious bodily harm^4^. This cognitive bias drives a detrimental cycle in which patients avoid painful but necessary movements to “protect” themselves, which in turn leads to physical deconditioning, heightened pain sensitivity, and poorer prognosis^5^.

For patients undergoing thoracoscopic lung surgery, activities like coughing and ambulation are crucial for preventing serious postoperative complications such as pulmonary embolism and infection, but often trigger pain^2^. Consequently, patients face a significant cognitive challenge involving the protective response to pain, which is interpreted as harm, versus the medical necessity to engage in painful recovery activities, which are essential for safety.

In this context, we propose the concept of “Threat Prioritization”—operationalized here as a patient’s cognitive evaluation where they weigh the competing threats of activity-induced pain versus inactivity-induced complications. This concept draws on Protection Motivation Theory, which posits that health behaviors are influenced by the perceived severity of and vulnerability to health threats^6^. According to goal-regulation frameworks, when multiple goals compete for cognitive resources, individuals prioritize the goal associated with the most salient perceived threat. In the postoperative context, the threat of pain and the threat of complications form a competitive relationship, and a patient’s threat prioritization may determine their behavioral pattern^7^.

This study aimed to examine the interplay between threat prioritization, FOP dynamics, and engagement in essential postoperative activities among patients undergoing thoracoscopic lung surgery. We formulated two hypotheses. First, patients prioritizing the threat of postoperative complications as greater than the threat of activity-related pain will demonstrate greater engagement in postoperative activities, such as coughing and ambulation. Second, postoperative FOP is dynamic and potentially modifiable, decreasing with engagement in activities where pain is manageable or perceived as tolerable.

## Methods

### Design and setting

This was an observational prospective cohort study. The study was conducted from January 23 to May 31, 2024, at the Thoracic Surgery Department of Wuhan Union Hospital, Tongji Medical College, Huazhong University of Science and Technology. The study was reviewed and approved by the Medical Ethics Committee of Wuhan Union Hospital, Tongji Medical College, Huazhong University of Science and Technology (Approval No. 20230987), and all methods were carried out in accordance with relevant guidelines and regulations along with ethical approval and consent.

### Participants

Eligible participants were adults (≥ 18 years) scheduled for video-assisted thoracoscopic lung surgery(VATS) under general anesthesia, without documented neurological or psychiatric disorders. Written informed consent was obtained from all subjects prior to their participation in the study. Exclusion criteria included conversion to open thoracotomy, postoperative Intensive Care Unit (ICU) transfer, unplanned early discharge, medical contraindications to early ambulation, or inability to cooperate due to communication barriers.

### Standard postoperative care and analgesia protocol

All participants received standard postoperative care, including intravenous Patient-Controlled Analgesia (PCA) for 72 h. For patients who limited or discontinued PCA use due to opioid-related side effects (e.g., nausea or dizziness)^8^, non-opioid alternatives such as Diclofenac Sodium were administered as rescue analgesia.

From postoperative day (POD) 1 through POD3, nursing staff provided daily guidance, educating patients on preventing complications through activity: “Regular coughing and walking soon after your surgery are very important. They help clear your lungs to prevent serious infections and keep your blood moving to prevent dangerous clots like pulmonary infection and deep vein thrombosis. While some discomfort is expected, moving is key to a safer and faster recovery.“^9^ Patients were instructed to cough hourly while awake and ambulate therapeutically (≥ 15 minutes continuously, ≥ 3 times/day) starting on POD1. While nurses provided encouragement, the actual frequency and duration of activity were largely patient-initiated.

### Outcome measures and data collection

Data were collected by trained research personnel or extracted from clinical records.

#### FOP

FOP was assessed on the morning of POD1 and POD3 using the validated Simplified Chinese version of the Fear of Pain Questionnaire-9^10^. This 9-item scale uses a 5-point Likert response (1 = never to 5 = always), yielding total scores from 9 to 45, with higher scores indicating greater FOP.

#### Activity-linked breakthrough pain (BTP) requests

Nurses documented whether patient requests for supplemental analgesia (BTP doses beyond PCA) between POD1 and POD3 were explicitly linked by the patient to coughing or ambulation. Data recorded the occurrence times of such activity-linked BTP requests.

#### Threat prioritization assessment

On the morning of POD3, patients responded to a single, standardized question: “Thinking about your recovery right now, what concerns you more: the pain you might feel when moving or coughing, OR the risk of developing complications like lung infections or blood clots if you don’t move or cough enough?” Responses categorized patients into “Pain-Focused” or “Complication-Focused” groups.

#### Cough performance

Assessed on the morning of POD1 and POD3. Patients sat upright and performed a forceful cough. A trained nurse, blinded to the patient’s threat prioritization response, rated cough strength using the Semi-Quantitative Cough Strength Score (SCSS)^11^, ranging from 0 (no cough) to 5 (strong, sustained cough).

#### Ambulation

Assessed based on activity logged through the end of POD3, verified via nursing documentation and brief patient interview on POD4. Total Count of Therapeutic Ambulation Episodes was defined as the cumulative number of distinct therapeutic ambulation episodes (each ≥ 15 min) from POD1 through POD3.

#### Clinical and safety outcomes

To assess safety and potential confounders, we collected data on surgery duration, intraoperative blood loss, chest tube duration, length of stay and the occurrence of postoperative air leaks (up to POD3). PCA Cessation Status was defined as the postoperative day (0–3) on which the patient discontinued PCA use. Pain intensity at rest and during movement was assessed on POD1 using a numerical rating scale (NRS, 0–10) to control for baseline pain differences.

## Statistical analysis

Descriptive statistics included means (standard deviations, SD) for normally distributed continuous variables and medians (interquartile ranges, IQR) for non-normally distributed data. Normality of data distribution was assessed using the Shapiro-Wilk test. Between-group comparisons were performed using the independent samples t-test for normally distributed continuous variables and the Mann-Whitney U test for non-normally distributed data. Categorical variables were analyzed using the Chi-square test or Fisher’s exact test (when expected counts were < 5). Paired t-tests were used to assess within-subject changes in FOP and SCSS from POD1 to POD3.

Multiple linear regression was employed to examine predictors of ΔFOP, calculated as FOP at POD3 − FOP at POD1, where negative values indicate greater fear reduction. Key demographic and surgical variables were retained in the model a priori. Multicollinearity was checked using Variance Inflation Factor (VIF). All analyses were performed using SPSS (version 26.0) and Python (Statsmodels), with statistical significance set at α = 0.05 (two-tailed).

## Results

### Participant characteristics

**Fig. 1:**
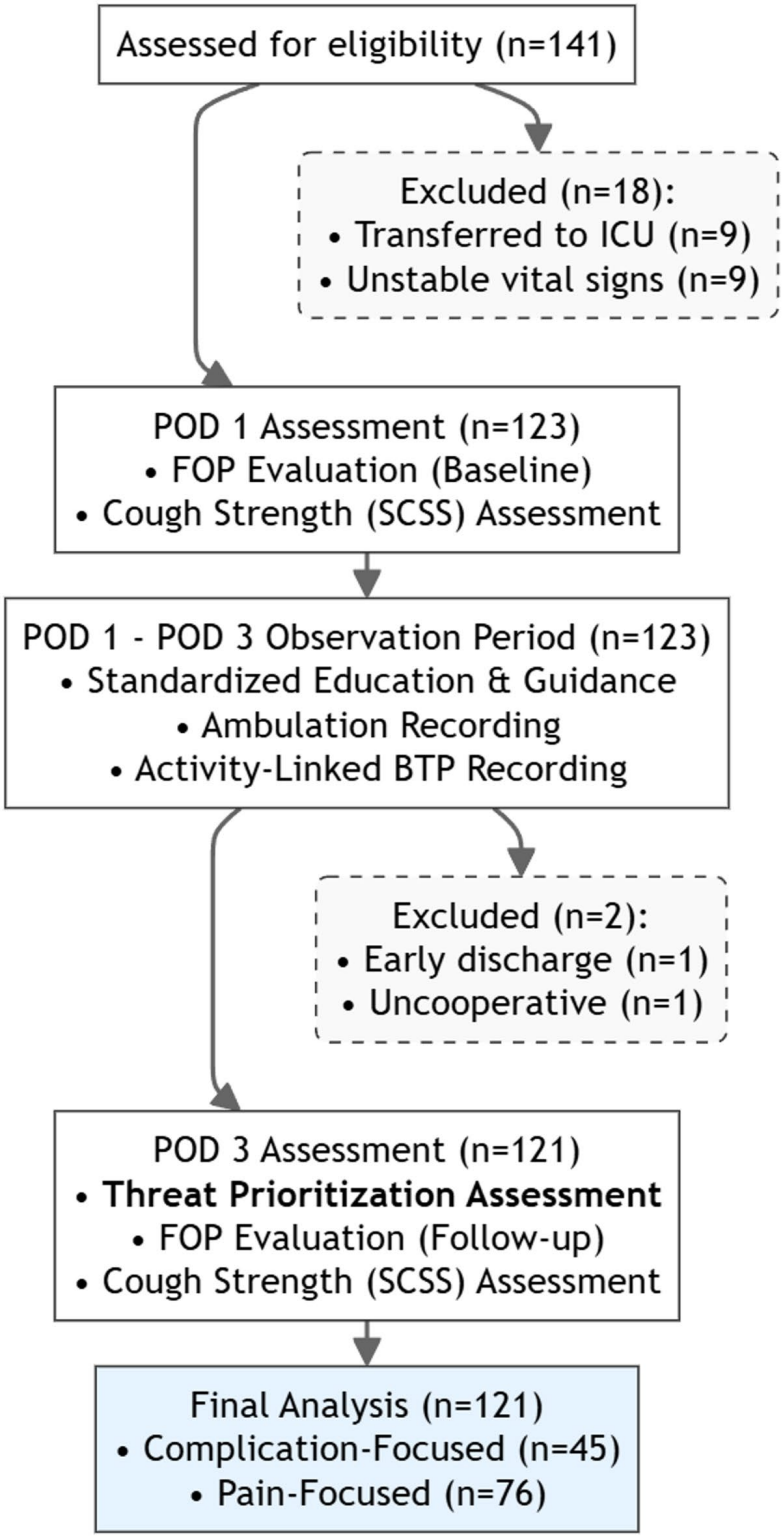
Study flow diagram. Note: ICU, Intensive care unit; FOP, Fear of pain; SCSS, Semi-quantitative cough strength score.

Initially, 141 patients met eligibility criteria. Twenty were excluded for the following reasons: nine required ICU transfer, nine had medical contraindications to ambulation (unstable vital signs), one was uncooperative, and one had an early discharge. The final analysis included 121 participants (Fig. 1). Based on the POD3 Threat Prioritization Assessment, 45 (37.2%) were categorized as Complication-Focused and 76 (62.8%) as Pain-Focused.

Baseline characteristics, including age, gender, body mass index(BMI), smoking history, and comorbidities, did not differ significantly between the groups (all *p* > 0.05). Surgical factors were comparable, with surgery method (*p* = 0.985), median surgery duration (95.50 vs. 100.00 min, *p* = 0.478), and median intraoperative blood loss (30.00 vs. 50.00 ml, *p* = 0.087) showing no significant differences. The prevalence of chronic obstructive pulmonary disease (COPD) was also similar between groups (8.9% vs. 11.8%, *p* = 0.765). Details are shown in Table 1.

**Table 1:** Baseline characteristics of participants by threat prioritization group (*n* = 121).

Variables	Complication-Focused (*n* = 45)	Pain-Focused (*n* = 76)	Statistic Value	*p*-value
Age (years), Mean (SD)	57.53 (9.58)	56.58 (9.72)	*t* = 0.525	0.601
Gender (Male), *n* (%)	16 (35.6)	28 (36.8)	*χ*² = 0.000	1.000
BMI (kg/m²), Median (IQR)	24.56 (20.28, 28.30)	23.99 (21.27, 28.48)	*U* = 1682.000*	0.883
Smoking History, *n* (%)	20 (44.4)	36 (47.4)	*χ*² = 0.015	0.902
Comorbidities, *n* (%)				
Hypertension	14 (31.1)	31 (40.8)	*χ*² = 0.757	0.384
Diabetes	10 (22.2)	18 (23.7)	*χ*² = 0.000	1.000
COPD	4 (8.9)	9 (11.8)	Fisher	0.765
Surgery Method, *n* (%)			*χ*² = 0.030	0.985
Wedge Resection	5 (11.1)	9 (11.8)		
Segmental Resection	21 (46.7)	36 (47.4)		
Lobectomy	19 (42.2)	31 (40.8)		
Surgery Duration (min), Median (IQR)	95.50 (84.00, 120.00)	100.00 (85.75, 125.00)	*U* = 1577.500*	0.478
Blood Loss (ml), Median (IQR)	30.00 (20.00, 50.00)	50.00 (20.00, 50.00)	*U* = 1404.000*	0.087
Pain at Rest (POD1), Median (IQR)	5.00 (4.00, 5.00)	5.00 (4.00, 5. 00)	*U* = 1758.000*	0.792
SCSS(POD1), Median (IQR)	2.00 (1.00, 2.00)	1.00 (1.00, 2.00)	*U* = 1945.500*	0.182

Note: SD, standard deviation; IQR, interquartile range; BMI, body mass index. * Mann-Whitney U test statistic reported.

### Hypothesis 1: Threat prioritization and postoperative activity

**Fig. 2:**
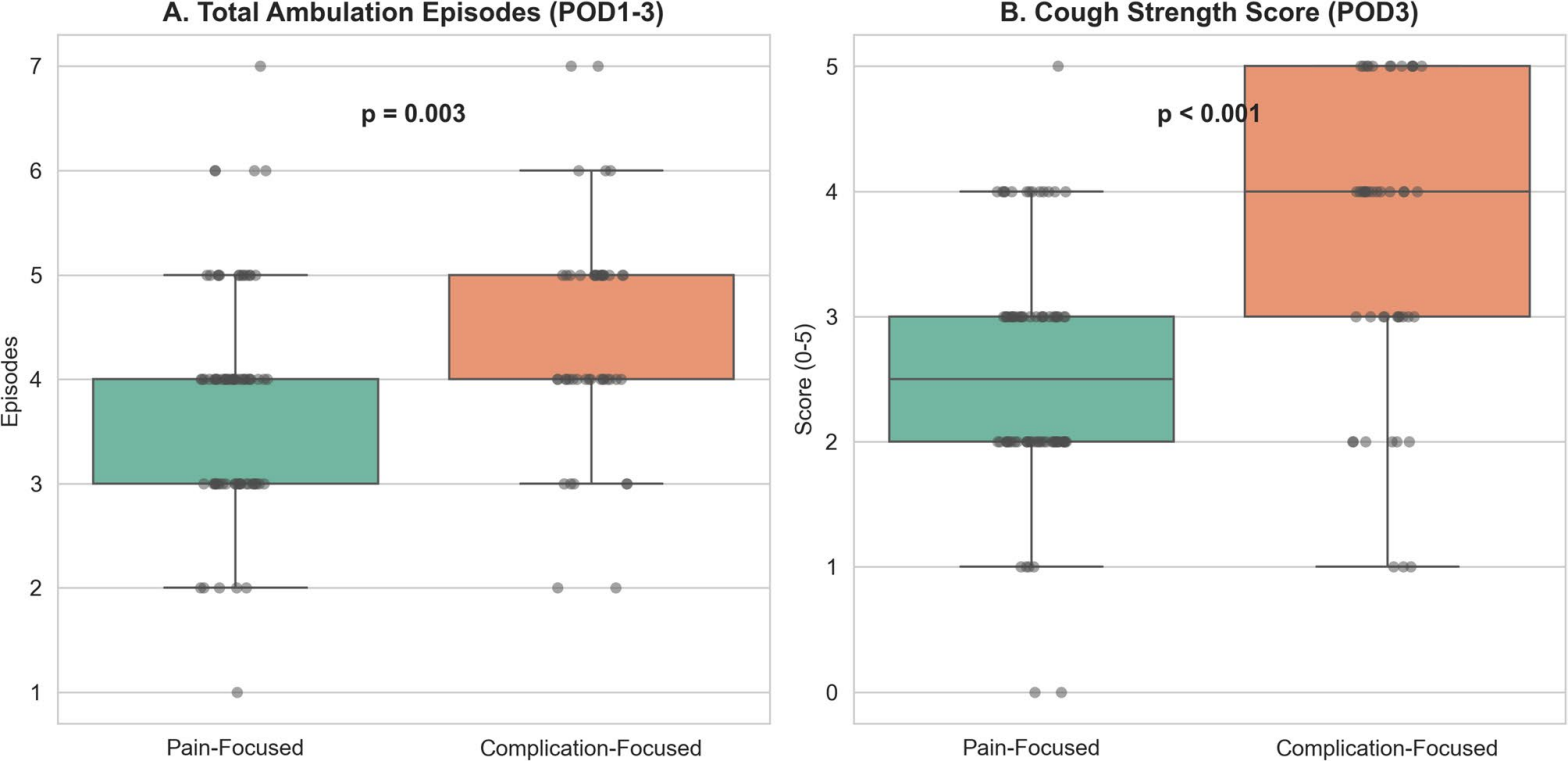
Comparison of postoperative engagement between threat prioritization groups.

We compared cough performance and ambulation between the Complication-Focused and Pain-Focused groups (Table 2). Baseline cough strength (SCSS) on POD1 did not differ significantly between groups (*p* = 0.182). However, by POD3, the Complication-Focused group showed stronger cough performance (Median SCSS 4.00 [IQR 3.00, 5.00] vs. 2.50 [IQR 2.00, 3.00], *p* < 0.001) and higher distribution of ambulation episodes (Median 4.00 [IQR 4.00, 5.00] vs. 4.00 [IQR 3.00, 4.00] episodes, *p* = 0.003) (Fig. 2).

Increased activity in the Complication-Focused group was not associated with a higher rate of postoperative air leaks (20.0% vs. 19.7%, *p* = 1.000), nor was it significantly associated with length of stay (Median 11.00 vs. 12.00 days, *p* = 0.200). These findings suggest that prioritization of activity over pain was not associated with increased perioperative morbidity in this sample.

**Table 2:** Comparison of postoperative activities and safety outcomes.

Variables	Complication-Focused (*n* = 45)	Pain-Focused (*n* = 76)	t / U / χ²	*p*-value	95% CI
SCSS (POD3), Median (IQR)	4.00 (3.00, 5.00)	2.50 (2.00, 3.00)	*U* = 2509.000*	< 0.001	(0.56, 1.41)
Ambulation Count, Median (IQR)	4.00 (4.00, 5.00)	4.00 (3.00, 4.00)	*U* = 2236.000*	0.003	(0.18, 0.98)
Air Leak, *n* (%)	9 (20.0)	15 (19.7)	*χ*² = 0.000	1.000	-
Length of Stay (days), Median (IQR)	11.00 (10.00, 14.00)	12.00 (10.00, 16.00)	*U* = 1472.000*	0.200	(-2.80, 0.31)

Note: SCSS, Semi-Quantitative Cough Strength Score; IQR, interquartile range; CI, confidence interval. Ambulation and Air leak data reflects occurrence up to POD3. * Mann-Whitney U test statistic reported.

### Hypothesis 2: Dynamic modification of FOP

Multiple linear regression was employed to examine predictors of the change in FOP (ΔFOP). Consistent with established methodological practice in regression modeling, we included age, sex, and surgical extent (wedge resection, segmentectomy, lobectomy) in the multivariable model a priori, as these are clinically established determinants of postoperative recovery, mobility, and respiratory function. Including these variables controls for potential residual confounding, even if they do not reach statistical significance in this dataset. Variables entered into the model included Ambulation Count, Activity-Linked BTP, Change in Cough Strength (ΔSCSS), Surgery Duration, Baseline Pain (POD1), PCA Cessation Status, Baseline FOP (POD1), Change in Pain Score (ΔPain), Age, Gender, and Surgical Extent (Segmentectomy, Lobectomy). The final model was statistically significant (*F*(12, 108) = 8.89, *p* < 0.001) and explained 43.3% of the variance in FOP reduction (Adjusted *R*² = 0.433, Table 3).

**Table 3:** Multiple linear regression predicting change in ΔFOP (*N* = 121).

Variables	B	Std. Error	Std. β	*p*-value	95% CI
Ambulation Count	-1.25	0.28	-0.36	< 0.001	(-1.80, -0.70)
Activity-Linked BTP	5.31	1.03	0.38	< 0.001	(3.27, 7.34)
Baseline FOP (POD1)	-0.14	0.05	-0.24	0.002	(-0.23, -0.06)
Surgery Duration	-0.009	0.004	-0.17	0.026	(-0.02, -0.001)
PCA Cessation (POD)	0.81	0.45	0.18	0.077	(-0.09, 1.70)
ΔSCSS	-0.38	0.20	-0.14	0.061	(-0.78, 0.02)
Gender (Male)	-1.10	0.87	-0.14	0.203	(-2.82, 0.61)
Baseline Pain (POD1)	-0.35	0.41	-0.10	0.395	(-1.16, 0.46)
Segmentectomy	0.59	0.90	0.08	0.511	(-1.20, 2.37)
Lobectomy	0.34	0.90	0.04	0.705	(-1.44, 2.12)
Age	-0.02	0.03	-0.04	0.598	(-0.07, 0.04)
ΔPain	-0.02	0.34	-0.01	0.964	(-0.69, 0.66)
(Constant)	7.94	2.57	-	0.002	(2.85, 13.02)

Note: Adjusted R² = 0.433; CI, confidence interval; B, unstandardized regression coefficient; Std. β, standardized regression coefficient. ΔFOP = FOP(POD3) - FOP(POD1) (negative values indicate reduction). ΔSCSS = SCSS(POD3) - SCSS(POD1). ΔPain = Pain(POD3) - Pain(POD1). BTP, breakthrough pain; SCSS, Semi-Quantitative Cough Strength Score; PCA, Patient-Controlled Analgesia. Gender was coded as 0 = female, 1 = male. Surgical extent was coded with wedge resection as reference category.

Multicollinearity was assessed using the Variance Inflation Factor (VIF). All predictors except the intercept showed VIF values below 3.5, with the highest being Pain(POD1) (VIF = 3.09) and ΔPain (VIF = 3.12), indicating no significant multicollinearity issues.

**Fig. 3:**
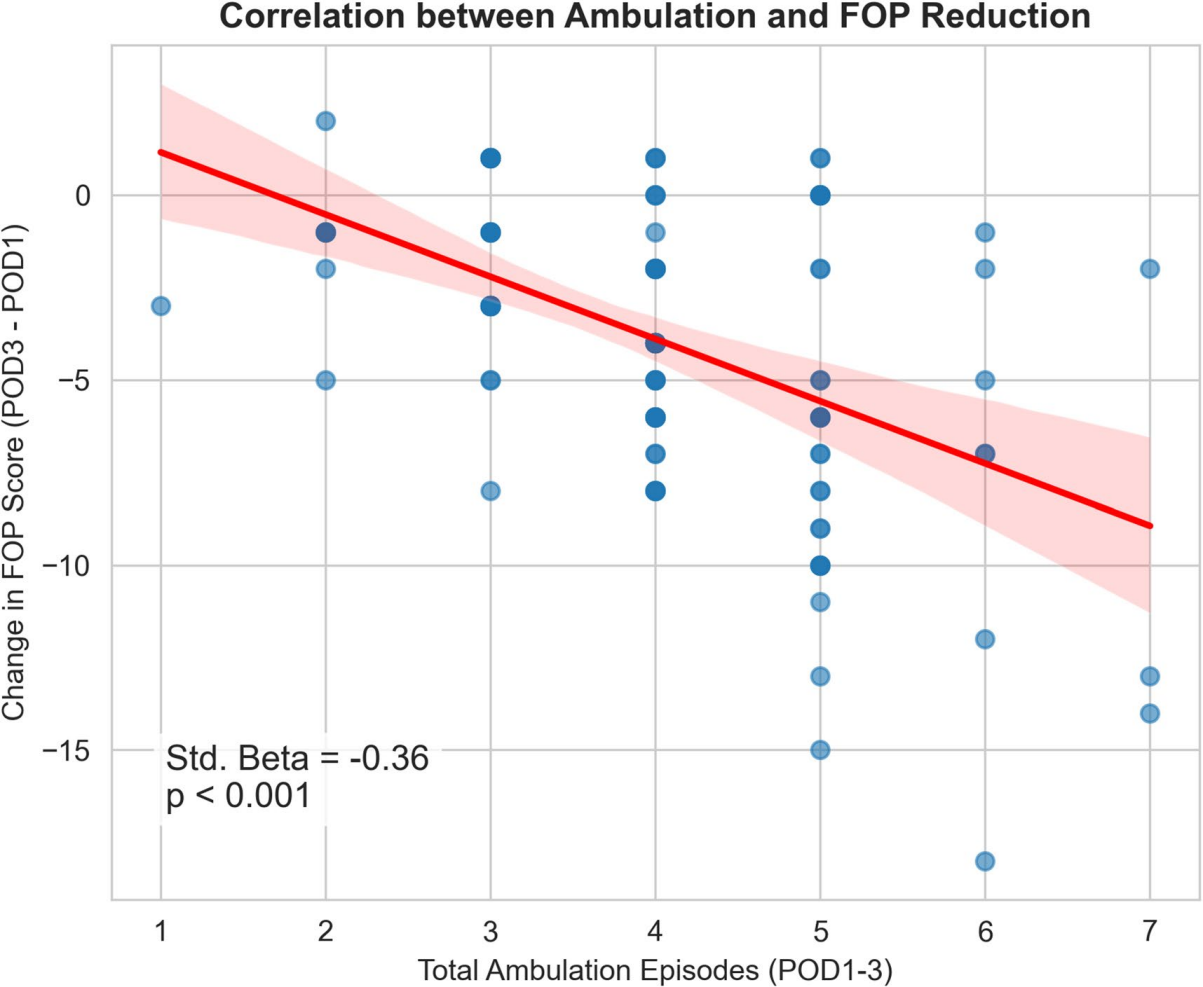
Association between Ambulation and FOP Reduction.

As shown in Table 3; Fig. 3, Ambulation Count (Std. *β* = -0.36) and Activity-Linked BTP (Std. *β* = 0.38) were the most strongly associated with FOP reduction, even after controlling for Baseline FOP. Baseline FOP was a significant predictor (*p* = 0.002), indicating regression to the mean. Physiological ΔPain was not a significant predictor (*p* = 0.964), suggesting that ΔFOP is associated with behavioral engagement rather than simple symptom relief. Age, gender, and surgical extent were included in the model a priori but were not significant predictors (all *p* > 0.05), suggesting that these variables did not substantially confound the relationship between activity engagement and FOP reduction in this sample.

Complication-Focused patients (*n* = 45) completed significantly more therapeutic ambulation episodes from POD1 to POD3 (*p* = 0.003) compared to Pain-Focused patients (*n* = 76). (B) Complication-Focused patients showed stronger cough strength on POD3 (*p* < 0.001). Center lines show medians; box limits indicate the 25th and 75th percentiles. Abbreviations: POD, postoperative day; SCSS, Semi-Quantitative Cough Strength Score.

Scatter plot with regression line (*N* = 121) showing a significant negative correlation between total ambulation episodes and the change in Fear of Pain (POD3 - POD1). Patients who ambulated more experienced a greater reduction in fear (Std. *β* = -0.36, *p* < 0.001). Abbreviations: FOP, fear of pain; POD, postoperative day.

## Discussion

The first finding from this study suggests that threat prioritization is associated with engagement in recovery-related health behaviors^12^. We observed that patients classified as “Complication-Focused” based on their POD3 self-report reported greater engagement in prescribed activities compared to those classified as “Pain-Focused”. Specifically, these patients showed higher cumulative ambulation counts over POD1-3 and stronger cough on POD3. This association suggests that a relative focus on mitigating serious non-pain threats could potentially play a role in reducing FOP-driven avoidance tendencies, consistent with models of attentional bias where focus shifts to the most salient perceived threat^13^. Consequently, our results suggest that a focus on competing consequences, as reported on POD3, is associated with greater activity engagement observed during the early postoperative period, which may relate to changes in pain expectations.

Secondly, the present findings suggest that FOP is dynamic and potentially modifiable in the early postoperative period. The decrease in FOP from POD1 to POD3 aligns with potential habituation or corrective learning^14^. Regression analysis showed that activity engagement, specifically more ambulation, was associated with greater FOP reduction. These findings align with exposure-based principles within the fear-avoidance framework, according to which confronting feared activities without catastrophic pain provides corrective feedback that may weaken the maladaptive movement = harm association^15^. Conversely, experiencing BTP explicitly linked to activity was associated with attenuated FOP reduction. This finding highlights the potential detrimental effect of poorly controlled pain during functional tasks, which may reinforce FOP and impede recovery progress^16^. Adequate analgesia, particularly timed around mobilization, may be important for translating activity into reduced fear.

A finding from this study is that baseline pain scores (POD1) did not differ significantly between groups (*p* = 0.792). This suggests that the difference in threat prioritization and subsequent behavior was not explained by the sensory magnitude of pain. Patients classified as Complication-Focused did not experience significantly less pain at baseline. Instead, this finding is consistent with a cognitive mechanism where the interpretation of the threat, such as Pain versus Complication, influences the behavioral response, independent of the nociceptive signal^17^.

Threat prioritization may involve the prefrontal cortex’s modulation of amygdala-mediated fear responses^18^. When patients shift attention from the threat of pain to the threat of complications, different neural pathways may be activated, potentially reducing hypervigilance to pain signals. This cognitive shift could engage top-down executive control processes that attenuate the affective dimension of pain perception. However, this hypothesis requires validation through future neuroimaging studies.

While our findings suggest an association between threat prioritization and activity engagement, patient safety should be prioritized. We observed a similar air leak rate in the Complication-Focused group, which was more active, compared to the Pain-Focused group (20.0% vs. 19.7%, *p* = 1.000) in our sample. While no major adverse events occurred, continued monitoring is warranted to confirm these findings in larger cohorts. Clinicians should balance mobilization goals with individual risk factors, potentially tailoring activity intensity for patients at high risk of air leaks. Regarding clinical significance, the Complication-Focused group achieved a higher cumulative therapeutic ambulation count over the 3-day period compared to the Pain-Focused group. While numerically modest, in the early postoperative context where many patients have difficulty initiating movement, this difference in behavior may be associated with cognitive reframing of fear.

Based on these findings, future interventions could explore the following strategies. First, preoperative threat cognition assessment may help identify patients at high risk for activity avoidance. Second, tailored health education could emphasize complication risks and the benefits of early activity, potentially shifting patients’ threat prioritization. Third, perioperative psychological interventions could assist patients in restructuring their threat cognition hierarchy. However, the effectiveness of these interventions requires validation through randomized controlled trials.

A major limitation of this study is the assessment of threat prioritization on POD3. This timing was chosen to avoid the priming effect, where early assessment might alert patients to the importance of complications and alter their natural behavioral responses. However, grouping patients based on their POD3 mindset to explain earlier behaviors (POD1-3) introduces the risk of reverse causality or post-hoc rationalization. It is possible that patients who mobilized more subsequently adopted a “complication-focused” attitude to justify their success, while those who were less active focused on pain. This rationalization may have occurred for physiological or other reasons. The present observational design demonstrates an association but cannot determine the direction of causality. Additionally, the single-item classification for threat prioritization is exploratory and warrants further validation. The single-center design limits the broad generalizability of our findings. Finally, the study may be underpowered to detect rare safety events, necessitating larger cohorts for confirmation.

## Conclusion

This study explores the concept of threat prioritization, grounded in Protection Motivation Theory, to explain differential engagement in postoperative recovery activities. Prioritizing the threat of complications over activity-related pain is associated with greater engagement in recovery activities after lung surgery. Postoperative FOP decreases, a process associated with activity participation but attenuated by activity-related pain. These associations appear to be mediated by cognitive appraisal rather than sensory input alone. While no statistically significant difference in air leaks was found, continued vigilance is warranted to confirm safety in larger samples. Future research should investigate preoperative assessment of threat cognition and targeted interventions to modify threat prioritization, with effectiveness evaluated through randomized controlled trials.

## Data Availability

The datasets used and analyzed during the current study are available from the corresponding author on reasonable request.
